# Physiopathological, Epidemiological, Clinical and Therapeutic Aspects of Exercise-Associated Hyponatremia

**DOI:** 10.3390/jcm3041258

**Published:** 2014-11-12

**Authors:** Caterina Urso, Salvatore Brucculeri, Gregorio Caimi

**Affiliations:** Department of Internal Medicine, University Medical Centre, 90127 Palermo, Italy; E-Mails: ursocat@gmail.com (C.U.); sasib@live.it (S.B.)

**Keywords:** hyponatremia, exercise, SIADH, hypertonic saline

## Abstract

Exercise-associated hyponatremia (EAH) is dilutional hyponatremia, a variant of inappropriate antidiuretic hormone secretion (SIADH), characterized by a plasma concentration of sodium lower than 135 mEq/L. The prevalence of EAH is common in endurance (<6 hours) and ultra-endurance events (>6 hours in duration), in which both athletes and medical providers need to be aware of risk factors, symptom presentation, and management. The development of EAH is a combination of excessive water intake, inadequate suppression of the secretion of the antidiuretic hormone (ADH) (due to non osmotic stimuli), long race duration, and very high or very low ambient temperatures. Additional risk factors include female gender, slower race times, and use of nonsteroidal anti-inflammatory drugs. Signs and symptoms of EAH include nausea, vomiting, confusion, headache and seizures; it may result in severe clinical conditions associated with pulmonary and cerebral edema, respiratory failure and death. A rapid diagnosis and appropriate treatment with a hypertonic saline solution is essential in the severe form to ensure a positive outcome.

## 1. Introduction

Exercise-associated hyponatremia (EAH) typically occurs during or up to 24 hours after prolonged physical activity, and is defined by a serum or plasma sodium concentration below the normal range of 135 mEq/L [1]. It is also reported to happen in individual physical activities or during organized endurance events carried out in austere environments, in which medical care is limited or often not available, and patient evacuation to definitive care is often greatly delayed [2]. Rapid recognition and appropriate treatment are essential in severe forms to ensure a positive outcome [2]; however, few randomized trials concerning EAH treatment have been reported [3].

EAH was first described in Durban, South Africa, in 1981; subsequently, Noakes *et al*. in 1985 described the occurrence of severe hyponatremia in four athletes who participated in endurance events that were longer than 7 hours [4]. EAH has been reported after sustained physical exertion during marathons, triathlons, and long-distance hikes, and by trekkers, climbers, and cold climate endurance athletes. Furthermore, it is likely that symptomatic or asymptomatic EAH is underreported in the literature [5,6,7,8,9,10].

## 2. Epidemiological Aspects

The described prevalence of EAH varies widely, in some measure because the diagnosis is based solely on abnormal biochemical results in an appropriate clinical setting. Many cases of EAH may be asymptomatic and are largely detected from blood samples taken from permitting athletes participating in research screening protocols; reported prevalence ranges from 0%–51% [2]. The highest prevalence of “asymptomatic” hyponatremia has been noted in ultra-marathon races covering 161 km in North America, in which the impact of EAH has ranged between 30% and 51% [7,10,11]. The prevalence of “asymptomatic” EAH is greater than that of “symptomatic” EAH; determinations of type rely on biochemical diagnosis of EAH in conjunction with clinical symptoms and signs [2]. Severe EAH results in a significant mental status change, which is caused by cerebral edema (exercise-associated hyponatremic encephalopathy, also defined as EAHE), and is sometimes associated with non-cardiogenic pulmonary edema [12]. Many studies confirmed that deaths can be directly attributed to complications associated with EAHE [13,14]. The overall prevalence of symptomatic EAH in all marathon participants is generally less than 1% [15], but the percentage of EAH observed in all symptomatic athletes seeking medical care has been reported to be higher than 23% in an Ironman Triathlon [16] and 38% in runners taking part in a marathon and an ultramarathon in Asia [17]. An increasing datum is that symptomatic EAH is now reported in much shorter distance events, such as half marathons [18] and sprint triathlons taking approximately 90 minutes [19]. Symptomatic cases of EAH have been reported with increased frequency in hikers and in the military. The described incidence of hyponatremia in Grand Canyon hikers seeking medical care from exercise-associated collapse or exhaustion was 16%, with an estimated impact rate between 2 and 4 per 100,000 persons. In 1993, clinically significant EAH in female hikers trekking through the Grand Canyon was first reported [5]. Since that initial case series, three separate case reports of symptomatic hyponatremia have been described in wilderness settings: the first involved a man trapped in a cold Alaskan environment [20], the second an athletic woman hiking at a low altitude in Nepal [6], and the third describes a physically fit man participating in an 8-day guided trek in New Guinea [21]. More severe cases have been reported by Spano *et al*. [22] in the Sierra Nevada Mountains of California and by Severac *et al*. [9].

US military services have reported an increased trend of EAH cases, primarily in the Marine Corps and army infantry personnel; there has only been one definite case of death [23,24,25].

Generally, in ultra-endurance athletes, the prevalence of EAH does not exceed 10% [4,26]. The prevalence of EAH seems higher in ultra-endurance (>6 hours) than in endurance races [27,28]; however, there have been variable results in studies investigating the prevalence of EAH in ultramarathons and other ultra-endurance events.

Knechtle *et al*. reported that the prevalence of EAH was no higher in ultra-endurance athletes compared to existing reports on marathoners and Ironman triathletes [29].

A study that investigated the prevalence of EAH in ultra-endurance athletes, such as ultra-mountain bikers (ultra-MTBers), ultra-runners, and mountain bikers (MTBers) in four races held in the Czech Republic, showed that 5.7% of the finishers developed post-race EAH with post-race plasma sodium of <135 mmol/L. The prevalence of EAH was higher in ultra-runners compared to ultra-MTBers [30]. Another study showed that EAH occurred in more than 50% of the finishers of a 161 km ultramarathon in California. Main outcome measurements of the study were pre-race and post-race body mass, total body water (TBW), extracellular fluid (ECF), and plasma sodium. Hyponatremia occurred in over half of the 161-km ultramarathon finishers but was not predicted by a change in body mass. The combination of pre-race TBW and percentage changes in TBW and ECF explained 87.5% of the variation in the incidence of hyponatremia [11]. Studies on EAH in ultra-running events in Switzerland [26] and New Zealand [31] reported a prevalence of 0% and 4%, respectively. The prevalence of EAH in ultra-MTBers (3.7%) and MTBers (7.1%) was also similar to studies of multistage MTB races in South Africa and in the Alps, in which fluid intake correlated negatively to race time [32,33].

Drinking behavior and the large amount of fluid available at the refreshment stations might give insights into why the prevalence of EAH is different in the different various disciplines [34]. It was observed that the faster athletes drank more than the slower ones [26,35].

Fluid intake was positively related to race performance and post-race plasma [Na^+^] was negatively associated with race performance [30].

Hyponatremic marathoners consume fluids at a higher rate (0.84 L/h) compared to non-hyponatremic runners. In mountain bikers, fluid intake was relatively high compared to marathoners, but this volume is within the hourly drinking volume limits of 0.8 L/h recommended for endurance exercise [13]. The high intensity of race, the difficult terrain, and the impossibility of drafting may be the main reasons that mountain bikers drink rather little compared to other ultra-endurance athletes [33]. The low mean fluid intake may explain a lower prevalence of EAH in ultra-MTBers [35].

A high prevalence of EAH was reported for open-water ultra-distance swimmers due to ingestion of water during the race [36] (Table 1).

**Table 1:** Prevalence of exercise-associated hyponatremia (EAH).

Disciplines	Subjects	Prevalence of EAH	References
Marathoners		up to 22%	[13,15,37,38]
Ultra-marathoners	Asymptomatic	30%–51%	[7,10,11]
	Athletes seeking medical care	38%	[17]
Mountain bikers		7.1%	[32]
Ultra-mountain bikers		3.7%	[33]
Ironman triathletes		1.8%–28%	[16,28,39]
Hikers		16%	[5,6,9,20,21,22]
Military		Indreased trend; one case of death	[23,24,25]
Swimmers	Males	8%	[36]
	Females	36%	

## 3. Physiopathology

**Figure 1:**
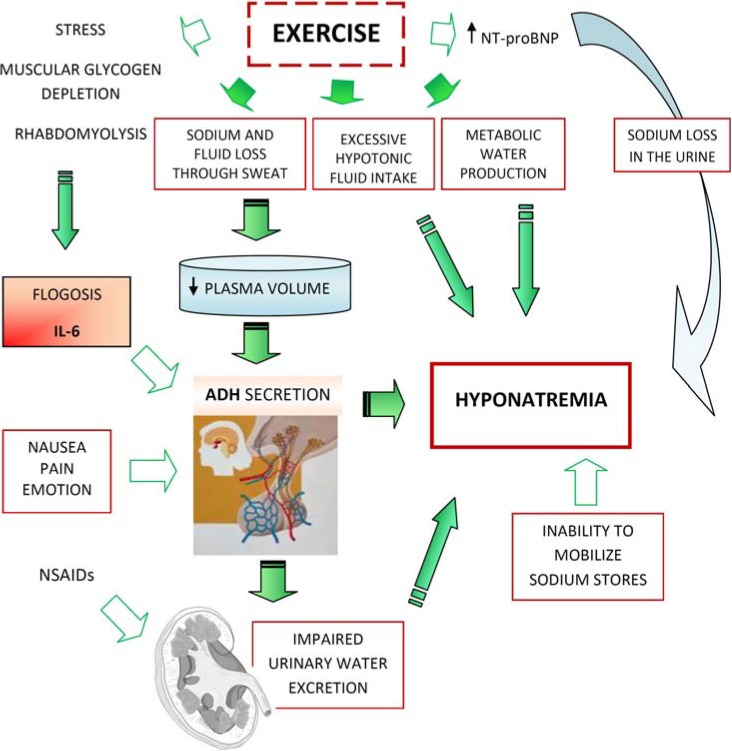
Physiopathology of EAH.

Two major mechanisms largely account for the development of EAH:

- Excessive fluid intake
- Impaired urinary water excretion, largely as a result of persistent secretion of antidiuretic hormone (ADH) (Figure 1).

Excessive fluid consumption leading to weight gain is considered the principal cause of reduced plasma sodium, and previous studies in ultra-endurance events have shown an association between fluid intakes, changes in body mass, and plasma sodium. Over-hydration appears to be the principal risk factor for the development of EAH. This occurrence reflects upon the weight gain observed in the majority of athletes who become hyponatremic. Evident sodium and fluid loss through sweat occur with prolonged exercise, leading to a decrease in extracellular fluid volume (ECFV) and plasma volume. The “effective” plasma volume further decreases with the redistribution of blood to the exercising muscles (which is caused by metabolic, hydrostatic, and haemodynamic mechanisms) [40].

The issue of whether sweat sodium loss contributes to the development of EAH remains controversial. The concentration of sodium in sweat varies widely, but it is usually between 15 and 65 mEq/L, and the volume of sweat varies from 250 mL/h to >2 L/h [41,42]. Although one might expect that hypotonic fluid losses from sweat would produce a rise in serum sodium, the development of hyponatremia following this occurrence has been attributed to hypotonic fluid replacement greatly exceeding fluid loss through sweat. The excessive concern over dehydration and hyperthermia may lead to excessive fluid intake among competitors [7,11].

This tendency toward aggressive hydration is illustrated by comparing the 1970 study documenting a rise in plasma sodium in eight runners who each consumed 500 mL of water to the 1985 study in which four runners ingested an average of 9 L of fluid over the same distance and experienced a decrease in plasma sodium to below 125 mEq/L [4,43]. Moderate intake of a carbohydrate clearly has been demonstrated to improve performance [44]. Although vigorous hypotonic fluid replacement blunts the decrease in plasma volume with exercise, a preferential expansion of intracellular fluid volume through osmotic equilibration leads to a relative total body water excess despite plasma volume depletion [45].

The overdrinking behavior seems to be supported by companies selling sports drinks. Moreover, these sports drinks (solutions enriched with mineral salts and ions, slightly less hypotonic than mineral water) do not prevent EAH [46].

Subjects with normal renal function and a regular diet can excrete about 500–1000 mL/h of water [47]. With the additional non-renal losses of water due to sweat and insensible fluid losses, athletes should be able to consume as much as 1000–1500 mL/h before developing water retention and dilutional hyponatremia. Thus, although fluid ingestion is necessary for the occurrence of EAH, it is not likely to be the only factor, except in those circumstances in which water intake is excessive (*i.e.*, >1500 mL/h) [2]. Under normal circumstances, ingestion of excessive water should suppress ADH, leading to the production of diluted and high-volume urine (where urine has an osmolality as low as 50 mOsm/kg and a volume of 500–1000 mL/h). The failure to suppress ADH reduces the ability of the kidneys to excrete a water load. The available data support the concept that many athletes with EAH have submaximal suppression of ADH and an inappropriately high urine osmolality; this is similar to SIADH. There are several nonosmotic stimuli that lead to the secretion of ADH that may be operable in endurance athletes: intense exercise itself, nausea or vomiting, hypoglycemia, and nonspecific stresses, such as pain and emotion. The excess of ADH production is responsible for potentially significant hyponatremia and is recognized as a key factor for EAH [48,49,50,51].

Moreover, it has been suggested that cytokine release (IL-6) during muscular glycogen depletion could also be involved in the nonosmotic stimulation of ADH. The hypothesis that IL-6 is involved in the pathogenesis of EAH has been confirmed in multiple studies [52]. In a rat sepsis model, IL-6 has been shown to reduce the expression of aquaporin-2, the target of ADH and ultimate regulator of water diuresis. A genetic predisposition to EAH, or severity of expression, may be related to single nucleotide polymorphisms of the promoter region for the IL-6 gene, as postulated in other inflammatory diseases [53]. Furthermore, prolonged endurance exercise induces an immune response, inflammatory and oxidative stress, as suggested by the evidence of an increase in levels of GSSG (oxidized glutathione) [54]. In marathon runners, 24 hours after the race, there is an increase in their white blood cell count with neutrophilia; these runners experience an increase of approximately 30 times normal levels of IL-6 and about 20 times normal levels of CRP. The levels of IL-6 increase exponentially with the intensity and duration of exercise; as does rhabdomyolysis and the consequent increase in serum CK levels correlated with IL-6 levels [55].

Other factors may lead to hyponatremia in endurance athletes. In a study of endurance athletes running for an average of 6 hours with ad libitum fluid intake, it was noted that even with a mass loss of 3.8 kg, plasma sodium maintained its normal levels [46]. Despite the loss in plasma volume in these subjects, there were significant increases in brain natriuretic peptide levels (NT-BNP). These elevations may lead to excessive losses of urine sodium and raise the risk of hyponatremia [56].

A possible mechanism for the maintenance of a normal serum sodium level despite weight gain is the release of sodium from internal stores [49]. Up to 25% of the body’s sodium is bound in bone and, although not osmotically active, is potentially recruitable into an osmotically active form [57]. Thus, this pool could minimize the decrease in plasma sodium induced by over-hydration or, if not mobilized, exacerbate hyponatremia [58].

The absorption of water retained in the gastrointestinal tract at the end of a race has been suggested as a cause for an acute drop in plasma sodium concentration. This may account for a transient lucid period after finishing a race followed by the acute development of cerebral edema within about 30 minutes after the competition [14].

In a study that reviewed medical records from marathoners (all participants in the 1998 Suzuki Rock “N” Roll Marathon), hyponatremic patients were compared to other runners with regard to race time, gender, clinical signs of dehydration, and use of NSAIDs. The study showed that hyponatremic runners reported drinking “as much as possible” during and after the race; they were less likely to have clinical signs of dehydration. An inverse relationship between initial plasma sodium and time of presentation was found, with a late presentation that predicts lower plasma sodium [45].

The breakdown of glycogen into smaller, more osmotically active molecules, such as lactate, during exercise initially increases cellular osmolality and shifts water into cells, leading to a rise in serum sodium. This may then be reversed within 5 minutes after the cessation of exercise, leading to a transient reduction in plasma sodium [59]. Variations in potassium balances that serve as effective osmoles may also affect the plasma sodium so that hypokalemia will lead to exacerbating hyponatremia [60]. Although the major risk factor for developing EAH is excessive water intake beyond the capacity for renal water excretion, risk factors for EAH also include low pace racing, prolonged exercise with a duration of more than four hours, low or high body mass, pre-exercise hyperhydration, the use of non-steroidal anti-inflammatory drugs (NSAIDs), and extremely hot or cold environments [16].

In marathoners, Mettler *et al*. noted an association between change in body mass and change in post-race plasma sodium [37]. In 161 km ultra-marathoners, however, Lebus *et al*. [11] did not find any association between changes in body mass and changes in plasma sodium. Also, in these ultra-marathon runners, the change in body mass showed no association with post-race plasma sodium or with a change in plasma sodium. This finding is not in agreement with the recent findings in marathoners reported by Mettler *et al*. [37]. These authors demonstrated an association between post-race plasma sodium and post-race plasma osmolality, and they supposed that the increased plasma osmolality might be due to the increased activity of ADH [61].

It seems that females are more susceptible to developing EAH, in particular severe hyponatremia, and they may be more symptomatic with equivalent levels of hyponatremia [12,62,63].

In rats, estrogen blunts the drive to consume sodium, with salt deprivation, and it induces renal oxytocin receptor mRNA synthesis and affects osmoregulation. Estrogen and progesterone inhibit the function of the Na^+^-K^+^ ATPase [64,65,66].

In humans, pregnancy hormones lower the thirst threshold, contributing to hypotonic volume expansion, while estrogen increases ADH secretion in post-menopausal females [67].

The gender effect may also produce a different behavior, such as more pressing adherence to hydration advice, during exercise or during longer exercise times. Although the incidence of women with symptomatic hyponatremia seems to be greater than that of men, when adjusted for BMI and racing time, the gender difference has not been shown to be statistically significant [38].

Along with other nonosmotic stimuli to ADH secretion, NSAIDs have been implicated as a risk factor in the development of EAH by potentiating the water retention effects of ADH in the kidney [39,68]. Inhibition of renal prostaglandin synthesis due to NSAIDs has deleterious effects on hemodynamic and renal function. Prostaglandins actually antagonize the effect of ADH and modulate renal salt and water excretion [14,69,70]. However, there is conflicting data, and further investigation is necessary to determine whether NSAID treatment, with respect to both classification and dosages, is a sure risk factor for the development of EAH. Other drugs associated with SIADH, such as selective serotonin reuptake inhibitors, may also increase the risk of EAH, but up to now, the data is not conclusive [2].

Exercise may also lead to rhabdomyolysis. This event has been associated with the use of diuretics, psychogenic polydipsia, and extreme exercise with water intoxication [71,72]. Subjects with cystic fibrosis may be at increased risk of rhabdomyolysis during exercise, considering their propensity for dehydration and hyponatremia by sodium loss via sweat through a defective chloride ion transport channel, the CF transmembrane conductance regulator (CFTR) [73]; in a recent study, CFTR mutations were found not to be associated with the development of EAH [74].

## 4. Clinical Aspects

Signs and symptoms of EAH include nausea, vomiting, confusion, headache, seizures, and it may result progressively, together with severe clinical conditions associated with cerebral edema, in brainstem compression, pulmonary edema, respiratory failure, and even death. Symptomatic EAH should be defined qualitatively in two subgroups: “mild” or “severe” differentiated by the presence or absence of neurologic manifestations. Although the early symptoms of EAH may be nonspecific, the presence of altered mental states, comas, seizures or respiratory distress indicates exercise-associated hyponatremic encephalopathy (EAHE) and should be promptly recognized. An interesting report underlined the importance of maintaining a broad differential diagnosis when evaluating a subject with altered mental status in an alpine setting. Despite an initial presumed diagnosis of altitude sickness, after an exact diagnostic evaluation, the symptoms appeared to be related to symptomatic hypotonic hyponatremia [22]. Symptomatic EAH in an endurance athlete may be confounded with profound dehydration, necessitating intravenous rehydration. The severity of EAH establishes fluid choices; the two categories reflect different intravenous fluid treatment options. Bennett reasonably points out that dehydration may be misdiagnosed as EAH. The Wilderness Medical Society practice guidelines affirm that administration of isotonic fluids could be “disastrous” in athletes with EAH; it may actually worsen hyponatremia, and may have potentially “devastating” consequences [2].

## 5. Prevention and Treatment

The primary strategy in preventing EAH is to avoid overdrinking during a race. Fluid ingestion, based on the sensation of thirst, during a race seems to be a prevention strategy because it reduces the risk of dehydration and over-hydration. Another strategy that has been shown to reduce the incidence of hyponatremia during endurance events is to reduce the availability of fluids along the routes of races (>3 km apart) [2]. In athletes who drink beyond thirst or fully replace 100% of body weight losses during a race, supplemental sodium may attenuate the reduction in plasma sodium concentration, but will not prevent the development of hyponatremia if overdrinking continues [75]. The monitoring of body weight change is a strategy commonly used in 161 km ultramarathons to prevent overhydration [7]. EAH has been reported with substantial weight loss in some environments, so weight loss should not exclude the diagnosis of EAH. Body weight can be monitored in organized events, and in the presence of weight gain during racing, fluid and sodium intake should be reduced until weight returns to between 2% and 4% of body weight loss from the baseline level [2].

The ingestion of sodium during exercise may be useful to performance by maintaining the plasma volume and/or by attenuating reduction in blood sodium. However, until now, the influence of sodium ingestion during a race or performance seems inconclusive [76].

Vrijens and Rehrer [77] showed improved time to exhaustion and attenuated plasma sodium drops with the ingestion of 61 mmol sodium (18 mmol L^−1^ solution), compared to a placebo drink (distilled water), during 3 hours of cycling in the heat. Anastaiou *et al*. showed that even small amounts of sodium (19.9 mmol/L; 39.8 mmol in total) ingested during three hours of racing in the heat were sufficient to attenuate the decrease in plasma sodium [78]. Similar findings were observed by Twerenbold *et al*. [79] during a four hour running time trial in temperatures ranging from 5–19 °C. Again, sodium ingestion (25 mmol h^−1^, 100 mmol total) resulted in a smaller decrease in the plasma sodium concentration from pre- to post-run measurements in female athletes. Conversely, Barr *et al*. reported no differences in the plasma sodium concentration at the end of 6 hours of racing in the heat, when either a water or saline solution was ingested. They therefore postulated that the reasons for the lack of differences between the two trials were due to changes in extracellular/intracellular fluid volumes or to the incomplete absorption of sodium by the intestine [80].

Two studies have also investigated sodium supplementation during Ironman races and both reported no differences between those taking sodium supplements and those without sodium supplementation [75,81]. A recent study aimed at investigating the effect of a sodium supplement on endurance performance during a 72 km road cycling time-trial in cool conditions did not show any effect on time-trial performance or plasma sodium. However, plasma sodium is influenced by fluid intake [82].

Appropriate management of EAH depends firstly on correctly diagnosing the condition. In fact, EAH can be mistaken for dehydration, heat illness, or acute altitude illnesses (Table 2). When EAH is considered in the differential diagnosis of a collapsed athlete and a point of care sodium concentration analysis is available, the field diagnosis of EAH becomes straightforward [2]. On-site analysis of plasma sodium concentration is not widely available, and large and established events often have no capacity for on-site blood analysis. If EAH is clinically suspected, an assessment of volume status should be considered before the treatment with intravenous fluids is carried out. An inappropriate intravenous fluid administration could result in worsening hyponatremia with potentially devastating consequences [2].

**Table 2:** Signs and symptoms of differential diagnosis (adapted from [2]).

Signs and symptoms	EAH	Heat illness	AMS	HACE or HAPE
Fatigue/weakness	+/−	+/−	+	+
Increased thirst	+/−	+	+/−	+/−
Temperature elevated	+/−	+++	−	−
Tachycardia	+/−	+	+/−	+/−
Nausea/vomiting	+/−	+/−	+/−	+/−
Headache/dizzines	+/−	+/−	+++	+++
Blurred vision	+/−	+/−	+/−	+/−
Confusion/disorientation	+/−	+/−	+/−	+/−
**Obtundation**	+/−	+/−	+/−	+/−
**Seizure**	+/−	+/−	+/−	+/−
**Coma**	+/−	+/−	+/−	+/−
**Respiratory distress**	+/−	−	+/−	+/−
Oliguria	+/−	+	+/−	+/−

EAH, exercise-associated hyponatremia; AMS, acute mountain sickness; HACE, high altitude cerebral edema; HAPE, high altitude pulmonary edema; +/−, Possible; +, likely; +++, present; −, not present; The clinical signs that justify transfer to an emergency unit are in bold.

The perception that heat exhaustion is caused by dehydration and that the levels of dehydration observed in endurance sports must be treated immediately with intravenous fluids has been termed the “dehydration myth” and has endured for over a decade. Nevertheless, in endurance athletes, there have been observed body mass losses of 8% or greater without clinical symptomatology or adverse consequences [7]. In most cases, such levels of dehydration are not dangerous and rarely require intravenous rehydration, if oral fluids can be tolerated. Furthermore, transient postural hypotension is common in endurance athletes receiving post-event medical therapy [83]. This is caused by lower extremity blood pooling (once the athlete stops moving) and the consequent impairment of cardiac baroreceptor reflexes. These athletes show lightheadedness, dizziness, or syncope, and have been managed for dehydration or hyperthermia by race event medical providers [83].

A high clinical supposition of EAH requires fluid restriction and salt supplementation. However, fluid restriction is contraindicated in the case of dehydration and rhabdomyolysis (with impending acute kidney injury). Intravenous hypertonic saline (HTS) is an appropriate approach in suspected EAH with neurological deterioration, whereas an oral hypertonic saline solution would be an appropriate approach in suspected mild EAH. When possible, urine analysis for sodium and osmolality, and blood analysis for osmolality, should be obtained before the start of treatment [2]. Subjects with EAH who are neurologically stable can be advised to limit fluid intake and consume salty snacks, soups, or a small volume of hypertonic fluid until the onset of diuresis. Oral hypertonic saline solutions are an appropriate intervention in subjects with EAH when oral intake is possible. If the subject is unable to tolerate oral intake, or when there is no improvement, or when symptoms worsen with this therapy, the recommended treatment is a 100 mL bolus of 3% hypertonic saline infused through a peripheral vein in less than 60 seconds. If the initial treatment does not improve the patient’s condition, sodium level and clinical assessments should be performed to identify signs of cerebral and/or non-cardiogenic pulmonary edema. These are the key factors that will determine whether a transfer to an emergency unit for urgent treatment is required. Organized endurance races that do not have an on-site opportunity for the measurement of plasma sodium concentration and treatment with hypertonic saline should prearrange an appropriate emergency transport system [2].

**Figure 2:**
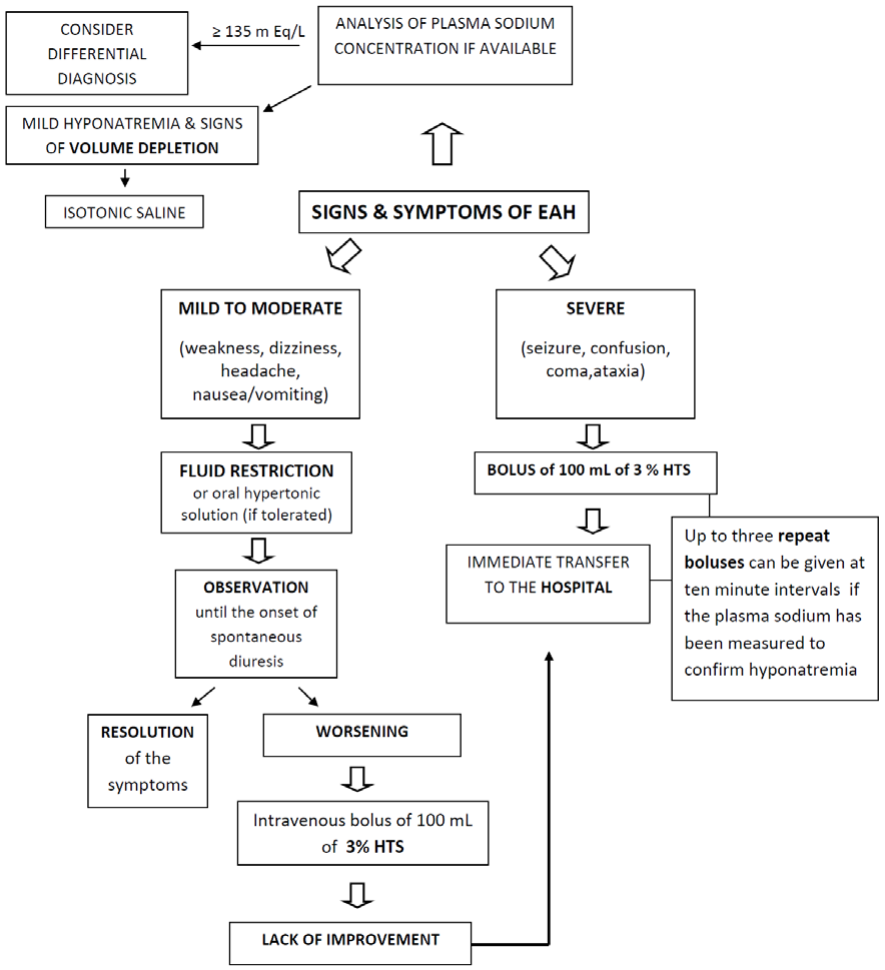
Algorithm for treatment of EAH. EAH, exercise-associated hyponatremia; HTS, hypertonic saline.

In subjects with suspected EAH, and especially in those with an altered mental state, sodium estimation should be obtained as rapidly as possible after arriving at the hospital. The intent of field management is to stabilize the subject until their management can be transferred to a definitive care medical center [12]. Treatment of serious EAH involves the administration of a 3% HTS at 1 mL/kg/h, that is subsequently adjusted according to the sodium status. Increases of sodium to 1 mmol/L/h during the first 6 hours, 9 mmol/L during the first 24 hours, and 18 mmol/L during the first 48 hours are acceptable. Ideally, sodium levels should not exceed 20–25 mmol/L during the first 48 hours [84] (Figure 2).

Small-volume boluses of intravenous HTS are the recommended therapy for exercise-associated hyponatremic encephalopathy (EAHE). Failure to properly diagnose and treat EAHE has been associated with significant morbidity and death. Current consensus statement guidelines recommend up to three 100/mL boluses of 3% HTS spaced at 10 minutes intervals to correct symptoms [2]. Advice is unclear regarding the maximal volume that can be safely administered in a given time period beyond these initial boluses. Data from previous literature suggest that the majority of subjects showed symptom resolution with these initial boluses [51]. However, use of large volumes of 3% HTS, from 600–950 mL, have produced no reported adverse outcomes [85,86].

Several reports demonstrated the potential adverse consequences of intravenous, normotonic solution hydration and the benefits of HTS for athletes with EAH. Another case report provides further support for our concern about the potential dangers of intravenous normotonic solution hydration in EAH [4]. In a report [87] regarding two overhydrated ultramarathon runners with symptomatic EAH, one received an intravenous normotonic solution and was hospitalized for 5 days, including for 36 hours in a semicomatose state. The other received intravenous 3% HTS and was fully alert within 3 hours and discharged after 8 hours. Davis *et al*. [45] describe a retrospective and prospective analysis of EAH treatment. The retrospective analysis involved 11 overhydrated marathon runners with severe EAH treated initially with intravenous normotonic solution; five of these runners (plasma sodium concentration range of 119–121 mEq/L) required hospitalization and three required intubation as well. Two of these subjects ultimately received HTS in the intensive care unit. The prospective trial involved four cases of overhydrated marathon runners with severe EAH (plasma sodium concentration range of 117–123 mEq/L) treated with 3% HTS; none required hospitalization, and the rate of plasma sodium correction with HTS was shown to be more rapid than for those runners who were treated with a normotonic solution [45]. Siegel *et al*. describes four marathon runners who became unresponsive, as if suffering from EAH; the two subjects that were treated with intravenous normotonic saline died with evidence of cerebral edema on postmortem examination. In contrast, in the two runners who received 3% HTS, the treatment caused a rapid neurologic improvement without adverse effects [48].

If neurologic impairment is present, HTS is the most effective agent and NS should be avoided. Subjects with EAH encephalopathy have been shown to recover more quickly when treated with 3% HTS *versus* isotonic saline and have a significantly reduced morbidity and mortality rate [48]. Furthermore, there is little data concerning oral hypertonic saline solutions. Considering whether or not it is useful to “asymptomatic” cases of EAH, the decision seems to be complicated by evidence that in clinical settings, the percentage of athletes with biochemical hyponatremia at the race finish who later progress to life-threatening hyponatremic encephalopathy and non-cardiogenic pulmonary edema is currently unknown. The efficacy of oral and intravenous 3% saline solution for treatment of “asymptomatic” clinical conditions needs further critical investigation. The clinical efficacy of an oral hypertonic solution in the treatment of EAH-induced delirium has been documented in three marathon runners [88]. In a large, randomized controlled trial set up to compare the efficacy of oral *versus* intravenous 3% HTS, in biochemical (non-neurological) cases of EAH, both oral and intravenous administration of a 100 mL bolus of 3% HTS are associated with a similar increase in plasma sodium without adverse consequences. The main physiological difference between the routes of administration was a significant plasma volume expansion with the intravenous (9%), but not the oral (1%) administration of HTS. It has been previously documented that intravenous 3% HTS elicits a greater plasma volume expansion compared to intravenous isotonic saline solution. Oral HTS is the intermediate treatment of choice for athletes diagnosed with EAH without significant symptoms [89].

## 6. Conclusions

Exercise-associated hyponatremia is still an underdiagnosed complication of endurance sports. EAH has a complex pathogenesis and a multifactorial etiology. Although hyponatremia is often mild and corrects itself without significant intervention, the development of neurologic sequelae suggests EAHE (altered mental status, seizures, coma, and death). Failure to rapidly diagnose and properly treat EAHE or pulmonary edema has resulted in death in otherwise young, healthy individuals.

Preventing EAH is the key factor in protecting participants in endurance events and other wilderness activities. There is a need to develop more rational fluid replacement strategies and education to optimize, rather than maximize, fluid intake during extreme exercise. Currently, there is no one recommendation that fits all individuals for fluid and salt consumption during endurance events, although prudent general guidelines include drinking to thirst and specifically avoiding excessively high fluid intake and monitoring body weight to avoid weight gain during a race. The serum sodium level and a rapid clinical assessment for signs of cerebral edema are the key factors that will determine urgent treatment with hypertonic saline solution.
